# Replacement of an Extracted Tooth

**Published:** 1859-10

**Authors:** Thos. H. Harding

**Affiliations:** Park-square, Regent’s-park


*Replacement of an Extracted Tooth.*

TO THE EDITOR OF THE LANCET—*Sir:* The belief is entertained by many individuals that when a sound tooth has been removed it cannot be returned to the alveolus, and resume its vitality. That this is incorrect, the following statement will prove, although I may mention that it is by no means the only instance which has occurred in my practice.

About two months ago, a gentleman came to me complaining of pain in the right side of his face, which appeared principally to affect the first upper molar tooth, which was to all appearances perfectly sound. He was anxious to have it extracted; this was declined, a liniment and aperient medicines being prescribed instead. He returned to me for the purpose of getting this tooth out, and I recommended him to go to the seaside for a fortnight, which he did, with decided benefit whilst there. On his return to town, however, the old pain came back, and at his most urgent request I removed the tooth, with the aid of the electrical anæthesia. It was, as I suspected, perfectly sound, and, after rinsing the mouth, in about five minutes it was replaced in the socket, in which it was kept by the teeth of the lower jaw coming in contact. He felt uncomfortable for a week; all uneasiness then passed away, and now the tooth is serviceable and sound.

The electric cautery, which has proved so useful in my hands was inapplicable to such a case as this, because there was no broken surface nor sign or irritation along the margin of the gums. My patient afterwards went under the care of Dr. B. W. Richardson, who treated him for gouty facial neuralgia with decided success.

I am, sir, yours respectfully,

THOS. H. HARDING.

Park-square, Regent's-park, 1859. [*Lancet.*]

